# P-1270. Surveillance of Antimicrobial Resistance Genes Detected Using BCID2 Among Veterans Affairs Medical Centers Within VISN 6

**DOI:** 10.1093/ofid/ofaf695.1460

**Published:** 2026-01-11

**Authors:** John Daniel Markley, Matthew M Hitchcock, Tristan Jones, Julia Ye, Angela Eckert, Daniel Tassone

**Affiliations:** Central Virginia VA Medical Center, Midlothian, VA; Richmond VA Medical Center, Richmond, Virginia; VCU Medical Center, Richmond, Virginia; Central Virginia VA Health Care System, Richmond, Virginia; Central Virginia VA Health Care System, Richmond, Virginia; Richmond VA Medical Center, Richmond, Virginia

## Abstract

**Background:**

Antimicrobial resistance (AMR) remains a top global health threat, accounting for 1.27 million deaths worldwide. Newer diagnostic tests have facilitated the rapid detection of genetic markers of resistance. However, regional surveillance of resistance genes remains largely untapped. The Veterans Health Administration (VHA), which has the largest integrated healthcare network in the U.S., offers a unique opportunity to analyze resistance gene data by region to better inform stewardship strategies. VISN 6 comprises 7 primary hospital sites in Virginia and North Carolina, serving more than 584,000 patients. All sites within VISN 6 utilize the BIOFIRE® Blood Culture Identification 2 (BCID2) Panel, which identifies 10 resistance genes from positive blood cultures. This study aims to describe resistance gene detection by region within VISN 6 in 2024.

**Figure 1:**
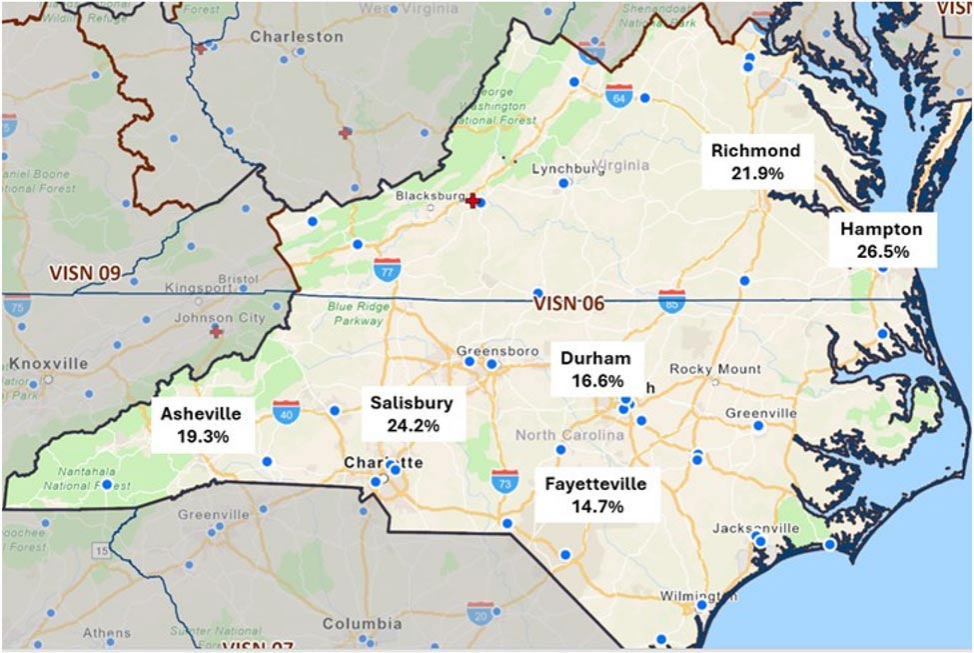
Variation in Proportion of BCID2 Tests Detecting an Antimicrobial Resistance Gene Among Health Care Centers in VISN 6

**Figure 2:**
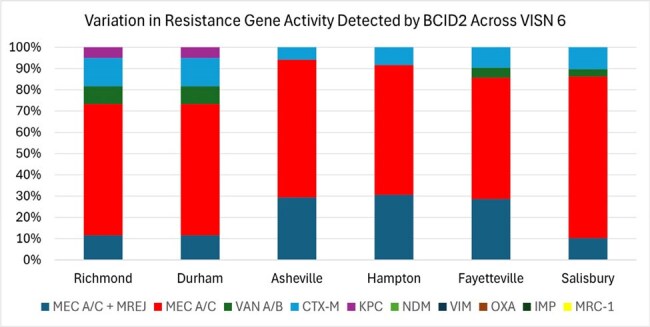
Variation in Resistance Gene Activity Detected by BCID2 Across VISN 6

**Methods:**

TheraDoc® was used to extract BCID2 results within VISN 6 for the calendar year 2024. Only one unique BCID2 result was included per patient per 7-day period. Organism and resistance gene detection data were collected and quantified by site location. ANOVA analysis was performed using Microsoft Excel (Version X, Microsoft Corporation, 2024).

**Results:**

A total of 1,095 BCID2 results were included in the analysis, with data available from six out of seven sites within VISN 6. Of these, 90.2% (988/1095) of BCID2 tests were positive for an organism. Across all available sites, 21% (204/988) of BCID2 tests identified an antimicrobial resistance gene. Mean resistance gene detection varied across sites, but was not statistically significant (p = 0.11). (Figure 1) Within sites, the proportion of resistance genes detected also varied. (Figure 2). *Staphylococcus aureus* harbored the Mec A/C and MREJ genes 43% (52/120) of the time. The CTX-M gene was detected in 13% (19/145) of *Escherichia coli* isolates. Only 1% (4/311) of *Enterobacterales* identified harbored a carbapenemase gene (4/4 *Klebsiella pneumoniae* carbapenemase, KPC).

**Conclusion:**

AMR gene detection using BCID2 varied by healthcare center across VISN 6. The most commonly identified resistance gene was Mec A/C, followed by Mec A/C + MREJ. Tracking and reporting AMR gene activity is critical for understanding trends in resistance over time.

**Disclosures:**

All Authors: No reported disclosures

